# Supplementary material for: Probing the topologically trivial nature of end states in antiferromagnetic atomic chains on superconductors
Source: Nat Commun. 2023 May 12;14:2742. doi: 10.1038/s41467-023-38369-w (PMC10182033; doi:10.1038/s41467-023-38369-w)
Supplement: Supplementary file 1 — Supplementary Information [file 41467_2023_38369_MOESM1_ESM.pdf]

Probing the topologically trivial nature of end states in antiferromagnetic atomic chains on superconductors

Supplementary Information

Lucas Schneider, Philip Beck, Levente Rózsa, Thore Posske, Jens Wiebe and Roland Wiesendanger

Correspondence to: jwiebe@physnet.uni-hamburg.de

Supplementary Note 1 | Calculations on the stability of states against local disorder

In Figs. 3a and b of the main manuscript text, the response of end states to local potential changes is studied. In the model introduced in Fig. 4 and in the Methods section of the main manuscript text, this type of disorder would correspond to disorder in the YSR energy E_0 at individual sites of the chain. Depending on the type of state that is perturbed by disorder, a different response is expected. In particular, the more local a certain state is, the more it is expected to react to disorder. To sketch this effect, we use the model presented in the Methods section of the main manuscript text and add randomly distributed values from the interval $[-\delta E_0, \delta E_0]$ to the on-site YSR energies $E_{0,i}$ for every site i of the chain. The results are shown in Supplementary Figs. 1a-d: for states perfectly localized on one individual site (Supplementary Fig. 1a), the spread in eigenenergies is – as expected – simply linearly increasing with the magnitude of the noise in the potential with slope of 1. In contrast, MMs are expected to be protected against disorder due to their non-local nature[1]. Supplementary Fig. 1b shows the same evolution of the eigenvalues with increasing disorder for a chain with N = 21 sites in the small-gap topologically non-trivial regime where the precursors of MMs (PMMs) still oscillate strongly in energy with increasing chain length due to large overlap of the Majorana wave functions[2]. Notably, the near-zero-energy mode is much more stable against local disorder than the atomic states in Supplementary Fig. 1a. However, this protection is not much stronger than the one from the finite-size quantized states[3,4] at finite energies, which also split only weakly for moderate disorder strength. This is a result of the spatially extended nature of both PMMs and the finite-size quantized states. In contrast, the MMs in Supplementary Fig. 1c, showing the large-gap scenario of the topologically non-trivial phase, are much more protected compared to all other states and remain at zero energy even for large values of δE_0 because of their clear nonlocality. Although the magnetic structure of the chain does not enter the minimal model explicitly, the parameter values chosen in Supplementary Fig. 1b and c can be thought of representing a ferromagnetic chain where the minigap is opened by the spin-orbit coupling. Finally, the phase with $t_2 >> t_1$ analyzed in Fig. 4 of the main manuscript text, modelling an antiferromagnetic chain, is studied in Supplementary Fig. 1d. The end states at finite energy of $E \approx \pm 0.5\ \mathrm{meV}$ react strongly to disorder. This is highlighted by the blue dashed lines in Supplementary Fig. 1d, indicating the (maximal) splitting of the states in Supplementary Fig. 1a, showing that the response of the end states is about 50% as strong as for the uncoupled sites. The effect can be understood to be a consequence of the strongly localized nature of the end states. Overall, these theoretical results agree with the experimental findings of Fig. 3 in the main manuscript text where the end states were found to split by several tens of μeV when perturbing them with magnetic or non-magnetic defects. It should be noted that they also split into two particle-hole pairs (i.e. four solutions in total) for every distribution of δE_0 whereas MMs and their precursors always split into a single particle-hole pair. This further supports the interpretation that our model maps well onto the experimental platform.

The perturbation of chains with additional YSR atoms in Figs. 3c and d is a slightly more complex situation, since this does not only shift the YSR energies in some of the chain atoms like hydrogen adsorption might do, but it rather couples additional energy levels to the chain. In order to incorporate this into our simulations, we study the system sketched in Supplementary Fig. 1e. This system consists of the same chain as in the previous panels now coupled via hopping terms t_+ to an additional site featuring energy levels at $\pm E_+$. This is the simplest implementation of an additional YSR atom in this model. We compute the resulting local density of states on the terminal sites of the chain on the left end, on the right end (both averaged over four sites) as well as on the additional site following Eq. (7) of the Methods section. The results are shown for three scenarios – the small-gap topologically non-trivial phase, the large-gap topologically non-trivial phase and the topologically trivial phase with finite energy end states – in Supplementary Figs. 1f-h. For the first case, i.e. the PMM phase, it can be seen that the zero-energy mode found for $t_+ << t_1$ remains at approximately zero-energy for a large interval of t_+. As t_+ is increased, the zero-energy state slightly splits in energy. But notably, this happens on both ends simultaneously, demonstrating the non-local nature of the PMMs. Furthermore, it can be seen that part of the PMMs' spectral weight is transferred onto the additional site. Supplementary Fig. 1g shows the results for the large-gap topologically non-trivial phase with MMs. Here, the zero-energy state remains robust on both ends even for large t_+. Just as found for the PMM phase, spectral weight of the MM moves laterally onto the additional site. Finally, we simulate the phase with dominant NNN hopping which is supposed to model our topologically trivial antiferromagnetic chains shown in the main manuscript text. Here, it can be seen that the left end's finite-energy end modes remain at a fixed energy of about ±0.5 meV whereas the end modes on the right end move to higher energies, thereby clearly demonstrating their local nature. Again, part of the spectral weight is located on the additional site for large t_+.

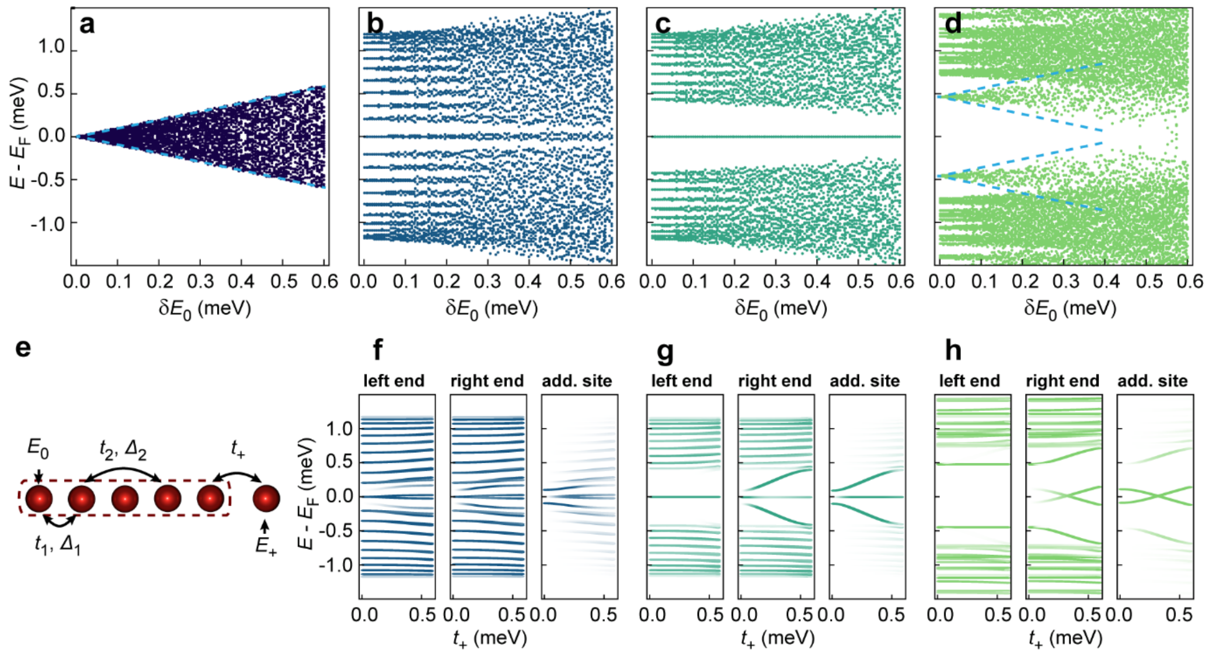

Supplementary Figure 1 | Stability of end states against local perturbations. a, Eigenstates of a chain of N = 21 uncoupled sites ($t_1 = t_2 = \Delta_1 = \Delta_2 = 0.0$ meV) with increasing potential noise δE_0 added to the on-site potential $E_0 = 0$ meV. Obviously, the distribution width of the eigenstates spreads linearly with a slope of 1 (blue dashed lines). **b**, Eigenstates of a chain of N = 21 sites in the small-gap topologically non-trivial regime vs. increasing potential noise δE_0. **c**, Large-gap topologically non-trivial regime. **d**, Phase with trivial end states in the regime $t_2 >> t_1$. Blue dashed lines represent the slope of 1 as also shown in panel a. **e**, Sketch of a chain with hopping elements analogous to the ones presented in the main manuscript text which is perturbed with an additional site on the right end. The additional site has its own on-site energy E_+ and is coupled locally to the right chain end via hopping of strength t_+. In the following panels, the chain is always N = 21 sites long. **f**, Spectral weight versus hopping t_+ in the small-gap topologically non-trivial regime evaluated on the left four sites, on the right four sites and on the additional site, respectively. **g**, Spectral weight versus hopping t_+ in the large-gap topologically non-trivial regime evaluated on the left four sites, on the right four sites and on the additional site, respectively. **h**, Spectral weight versus hopping t_+ for a chain with trivial end states in the regime $t_2 >> t_1$ evaluated on the left four sites, on the right four sites and on the additional site, respectively. Parameters for panels b and f: $t_1 = 0.6$ meV, $t_2 = 0.0$ meV, $\Delta_1 = 0.06$ meV. Panels c and g: $t_1 = 0.6$ meV, $t_2 = 0.0$ meV, $\Delta_1 = 0.2$ meV. Panels d and h: $t_1 = 0.0$ meV, $t_2 = 0.6$ meV, $\Delta_1 = 0.5$ meV. We set $\Delta_2 = 0.0$ meV and $E_+ = 0.1$ meV for all panels.

Supplementary Note 2 | Spin-polarized measurements on Mn/Ta(110)

The antiferromagnetic ground state of densely packed Mn chains along the $[1\bar{1}1]$ direction on Nb(110) was determined in Ref. 5 and confirmed by *ab-initio* calculations in Ref. 6. Yet, the magnetic couplings of Mn adatoms on Ta(110) are not *a priori* known. Therefore, we performed additional measurements with spin-polarized Cr tips[5,7] on Mn chains along $[1\bar{1}1]$ on Ta(110). Cr tips were made from high-purity Cr splinters glued into a W tip holder with conductive H20E glue[8]. The tip was subsequently heated to $T \approx 700$ K *in situ* and voltage pulses of 10 V were applied against a Pt(111) surface in order to remove oxide layers from the tip apex. Supplementary Fig. 2a shows an STM topography image of a Mn_9 chain on Ta(110) imaged with a Cr tip in a weak external field of $B_z = +20$ mT. Four maxima are observed on the chain, corresponding to the spatial positions of the atom No. 2, 4, 6 and 8. When reversing the field direction ($B_z = -20$ mT), imaging the same chain results in a pattern with three maxima on atoms No. 3, 5 and 7 and minima in between. These two images can be interpreted as follows: the Mn chain is antiferromagnetically aligned, yielding an alternating contrast on neighboring sites. While the Cr tip's magnetization is stable in an external field, the degeneracy of the two collinear antiferromagnetic Néel states sketched above the panels is slightly lifted by the external field acting on a structure with an odd number of sites[9]. The terminal chain sites always feature an electronic contrast which makes it hard to interpret the spin-contrast on them. This could be the reason why 3 and 4 maxima are found in the images instead of 4 and 5. Note that the spin-contrast is detected in the Z-signal since we use very small bias voltages of V_{stab} = 1 mV.

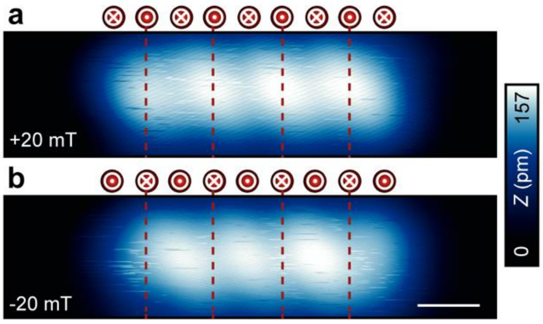

Supplementary Figure 2 | Spin-polarized STM images of the magnetic ground state of Mn chains on Ta(110). a, Constant-current STM image of a Mn_9 chain on Ta(110) in a weak out-of-plane magnetic field of $B_z = +20$ mT using a spin-polarized Cr tip with stable magnetization. The sketch above the image illustrates the antiferromagnetic spin texture of the nine atoms which are locked into one of the Néel ground states by the external field. **b**, Constant-current STM image of the same Mn_9 chain measured in a reversed field of $B_z = -20$ mT, revealing opposite spin contrast. Above the image, the reversed spins compared to panel a are sketched. Dashed lines are guides to the eye. The white scale bar corresponds to 500 pm. Parameters: V_{stab} = 1 mV, I_{stab} = 0.2 nA.

Supplementary Note 3 | Determination of tip and sample gaps

The numerical deconvolution process performed on the data presented in the main figures (see Methods for details) requires sufficient knowledge of the tip density of states $\rho_t(E)$. We assume a broadened Dynes density of states for the superconducting Nb tip apex, given by:

$$\rho_t(E) = \rho_0 Re\left[\frac{E - i\Gamma}{\sqrt{(E - i\Gamma)^2 - {\Delta_t}^2}}\right]. \qquad (S1)$$

Here, ρ_0 denotes the normal conducting DOS, *Re* is the real part, Γ is the Dynes broadening parameter and Δ_t is the tip's superconducting gap. Throughout this work, Γ = 0.01 meV is chosen for all tips used. The value of Δ_t is estimated by measurements of multiple Andreev reflections (MARs) appearing as weak additional peaks in d*I*/d*V* spectra for low junction resistances[10]. This effect is shown in Supplementary Fig. 3 for tunneling between a superconducting Nb tip and a clean Ta(110) sample surface: for high junction resistances (Supplementary Fig. 3a), the convolution of the sample's and tip's coherence peaks yields a large peak in d*I*/d*V* at $eV = \pm(\Delta_t + \Delta_{Ta})$, where Δ_{Ta} is the gap of the Ta sample.

In contrast, for lower junction resistances (Supplementary Fig. 3b), additional peaks at ± 1.25 mV and ± 0.64 mV are found. Since the latter value matches the superconducting gap of Ta while the first value is only slightly smaller than the gap of Nb, we attribute these peaks to Andreev tunneling occurring at $eV = \pm\Delta_t$ and $eV = \pm\Delta_{Ta}$, respectively[10]. Importantly, the MAR peaks are not visible in the high-resistance limit (Supplementary Fig. 3a), indicating that single-particle tunneling is the dominant transport channel and that the deconvolution process is justified. The result of a numerical deconvolution of the spectrum shown in Supplementary Fig. 3a is presented in Supplementary Fig. 3c. Indeed, this spectrum is well described by a Dynes density of states (see Eq. S1) as well when choosing a broadening parameter of Γ = 0.03 meV and a gap Δ_{Ta} = 0.64 meV.

The same characterization for measurements on the clean Nb(110) surface can be found in the Supplementary Information of Ref. 3.

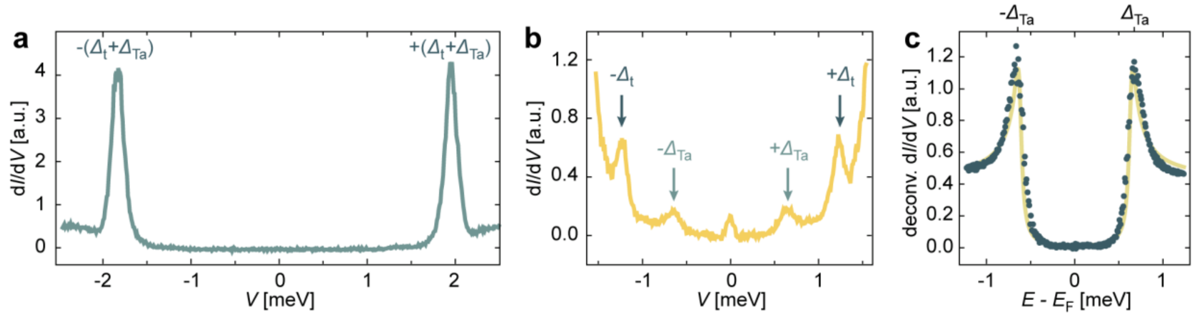

Supplementary Figure 3 | Determination of tip and sample gaps for measurements on Ta(110). a, d*I*/d*V* spectrum measured on the clean Ta(110) surface at high junction resistance (V_{stab} = -2.5 mV, I_{stab} = 1 nA, V_{mod} = 20 µV), showing prominent peaks at bias voltages $e \cdot V = \pm(\Delta_t + \Delta_{Ta})$ and no additional sub-gap peaks. **b**, d*I*/d*V* spectrum measured with the same tip at low junction resistance (V_{stab} = -2.5 mV, I_{stab} = 10 nA, V_{mod} = 20 µV) with distinct additional peaks visible at $e \cdot V = \pm\Delta_t = 1.25$ meV and $e \cdot V = \pm\Delta_{Ta}$ due to multiple Andreev reflection processes. Furthermore, Josephson tunneling occurs at zero voltage, yielding a zero-bias peak. **c**, Spectrum from panel a after numerical deconvolution (see Methods) using the previously determined tip gap parameter Δ_t from panel b. Assuming a superconducting density of states modeled by the Dynes function with $\Gamma = 0.03$ meV and $\Delta_{Ta} = 0.64$ meV (solid line) describes the data (dark points) well.

Supplementary Note 4 | Additional data measured on the perturbed Mn chain on Nb(110)

As it can be seen in Fig. 3a of the main manuscript, the defect on the left side of the Mn_{20} chain is the most prominent but not the only one on the surface near the chain. For instance, on the lower left side as well as on the upper right edge of the image, there are additional dark spots, which are absent in Fig. 3b, i.e., after applying voltage pulses. Therefore, we conclude that all these spots are due to mobile defects, which move out of the scan area once a bias pulse is applied. Thus, both end states on the left and right end in Figs. 3a and b slightly change their energy upon cleaning the area. Individual spectra recorded on the left and right chain end before and after pulsing, respectively, are shown in Supplementary Figure 4. It can be seen that the energies of the peaks on the chain's left end change much more visibly upon voltage pulsing than the ones on the right end. This is probably related to the stronger perturbation generated by the prominent defect on the left end (c.f. Fig. 3a) compared to the other mobile defects.

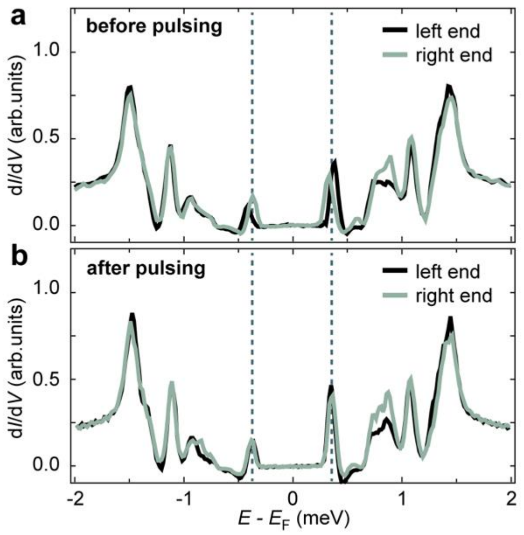

Supplementary Figure 4 | Individual spectra on perturbed Mn chains on Nb(110). a, Deconvoluted d*I*/d*V* spectra measured on both ends of the Mn_{20} chain shown in Fig. 3a. The left chain end is perturbed by a mobile defect, leading to an inequal energy of the end states on both sides. **b**, Deconvoluted d*I*/d*V* spectra measured on both ends of the Mn_{20} chain shown in Fig. 3b after applying local voltage pulses. The peaks are at approximately the same energy now. The vertical dashed lines are guides to the eye to better compare the energies of panels a and b. Parameters: V_{stab} = -6 mV, I_{stab} = 1 nA, V_{mod} = 20 µV.

Supplementary Note 5 | Additional data on the perturbed Mn chain on Ta(110)

In Figs. 3d-f of the main manuscript, we show individual spectra taken on a Mn_{22} chain on Ta(110) which is perturbed by an additional Mn adatom. We argue that the change in spectroscopy, in particular the presence and absence of a zero-energy state on the right end as the single atom's position is changed, is proof of the non-topological nature of the end state in the Mn chain. However, it could happen that the zero-energy end state merely shifts laterally to another position, e.g., further inside the chain as reported for hybrid Co-Fe chains on Re(0001) for instance[11]. We show full d*I*/d*V* line-profiles in Supplementary Figure 5 to demonstrate that this is not the case for the Mn chains on Ta(110). The zero-energy end state observed in Supplementary Figure 5a for example is absent in all other panels. This confirms that these end states are clearly local and thus topologically trivial in nature.

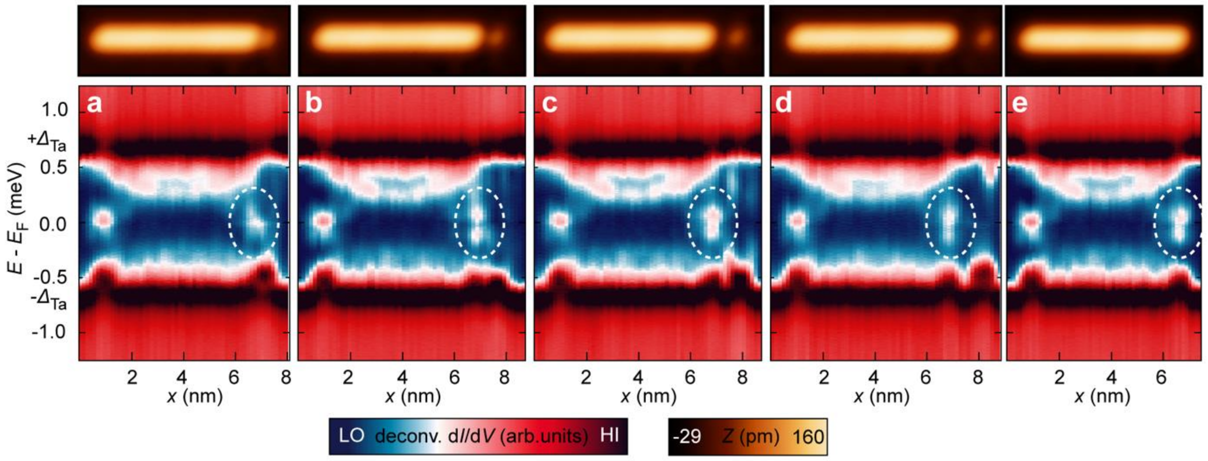

Supplementary Figure 5 | dI/dV line-profiles on perturbed Mn chains on Ta(110). a, Constant-current STM image of a Mn_{22} chain on Ta(110) and a single Mn atom located two lattice sites away from the right chain end (upper panel) and deconvoluted d*I*/d*V* line-profile measured along the longitudinal axis through the center of the ensemble. The lateral position of the line-profile is aligned with the topography above. **b**-**d**, Constant-current STM image and deconvoluted d*I*/d*V* line-profile with the single atom being located three (**b**), four (**c**) and five (**d**) lattice sites away from the right chain end. **e**, Constant-current STM image and deconvoluted d*I*/d*V* line-profile of the isolated Mn_{22} chain. STM image parameters: V_{stab} = -20 mV, I_{stab} = 0.2 nA. Line-profile parameters: V_{stab} = -2.5 mV, I_{stab} = 1 nA, V_{mod} = 20 µV. The dashed white ellipses mark the respective end states observed on the right end, which are subject to change as the single Mn atom is moved.

Supplementary Note 6 | Sub-gap quasiparticle interference measurements

The standing-wave-like patterns observed experimentally in Figs. 1b,d of the main manuscript can be explained by interference of energetically degenerate sub-gap quasiparticles with momenta $\boldsymbol{k}_\mathrm{i}$ and $\boldsymbol{k}_\mathrm{f}$ in the YSR bands[2,3]. As sketched in Fig 4e of the main manuscript, two dominant QPI branches with scattering vectors q_1 and q_2 ($|\boldsymbol{q}| = |\boldsymbol{k}_\mathrm{i} - \boldsymbol{k}_\mathrm{f}|$) are expected based on the minimal model of the main manuscript text for small E_0 (i.e., deep YSR states), $t_1 \ll t_2$ and $\Delta_1 \gg \Delta_2$ (appropriate for an antiferromagnetic chain). By performing a line-wise fast Fourier transform (FFT) analysis of the experimental data in Fig. 1d of the main manuscript, information about the YSR band dispersion can be extracted (Fig. 4g). Note that the FFT analysis does not directly display the band structure but the possible scattering vectors q in the QPI process.

Focusing on the hole-like part of the data in Fig. 4g ($E > 0$), two dominant arcs around $q = 0$ are observed, one with negative (supposedly q_1) and one with positive (supposedly q_2) curvature. Comparing these to the predicted scattering vectors in Fig. 4e and the theoretically simulated QPI pattern in Fig. 4f, it is found that q_1 is expected to be close to $q/2 = \pm\pi/4$ at the bottom of the hole-like band (indicated by the gray dashed lines), which is the case for the supposed q_1 branch extracted from the experimental data (Fig. 4g). Additionally, QPI from scattering of type q_2 (Fig. 4e) would result in a branch with positive effective mass, which could be the second arc marked in Fig. 4g. The electron-like part of the figure can be explained in the same way, although asymmetries in the particle and hole spectral weights of the individual YSR states[12,13] can lead to a slightly different appearance of the QPI patterns[3]. Interestingly, the end states at $\varepsilon_{+/-} \approx \pm 0.5\ \mathrm{meV}$ also feature a prominent Fourier component of $q/2 = \pm\pi/4$. Additional frequency components, e.g. at $\pm\pi/8$ or zero, can be attributed to artifacts of the FFT

acting on a peaked signal like an end state. This suggests that the end states inherit properties like the wavelength from the observed dispersive sub-gap band. A similar effect is known for topological MMs[14,15].

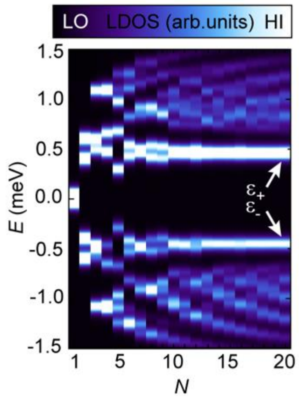

Supplementary Figure 6 | Calculated evolution of the end states with the length of the antiferromagnetic chains. Evolution of the LDOS calculated using the minimal model described in the main manuscript text on the terminal two sites of a chain with increasing number of sites *N*. The used parameters are $E_0 = 0.0$ meV, $t_1 = 0.1$ meV, $t_2 = 0.6$ meV, $\Delta_1 = 0.5$ meV, and $\Delta_2 = 0.0$ meV. For *N* = 1 and 2, there are no terminal sites and the total DOS is shown. The energies of the finite energy end states $\varepsilon_{+/-}$ are marked.

References

1. Awoga, O. A., Björnson, K. & Black-Schaffer, A. M. Disorder robustness and protection of Majorana bound states in ferromagnetic chains on conventional superconductors. *Phys. Rev. B* **95**, 184511 (2017).
2. Schneider, L. *et al.* Precursors of Majorana modes and their length-dependent energy oscillations probed at both ends of atomic Shiba chains. *Nat. Nanotechnol.* **17**, 384–389 (2022).
3. Schneider, L. *et al.* Topological Shiba bands in artificial spin chains on superconductors. *Nat. Phys.* **17**, 943–948 (2021).
4. Ben-Shach, G. *et al.* Detecting Majorana modes in one-dimensional wires by charge sensing. *Phys. Rev. B* **91**, 045403 (2015).
5. Schneider, L., Beck, P., Wiebe, J. & Wiesendanger, R. Atomic-scale spin-polarization maps using functionalized superconducting probes. *Sci. Adv.* **7**, eabd7302 (2021).
6. Lászlóffy, A., Palotás, K., Rózsa, L. & Szunyogh, L. Electronic and Magnetic Properties of Building Blocks of Mn and Fe Atomic Chains on Nb(110). *Nanomaterials* **11**, 1933 (2021).
7. Schlenhoff, A., Krause, S., Herzog, G. & Wiesendanger, R. Bulk Cr tips with full spatial magnetic sensitivity for spin-polarized scanning tunneling microscopy. *Appl. Phys. Lett.* **97**, 083104 (2010).
8. EPOXY TECHNOLOGY, INC. - 14 Fortune Drive, Billerica, Massachusetts 01821, USA.
9. Khajetoorians, A. A. *et al.* Atom-by-atom engineering and magnetometry of tailored nanomagnets. *Nat. Phys.* **8**, 497–503 (2012).
10. Ternes, M. *et al.* Subgap structure in asymmetric superconducting tunnel junctions. *Phys. Rev. B* **74**, 132501 (2006).
11. Schneider, L. *et al.* Controlling in-gap end states by linking nonmagnetic atoms and artificially-constructed spin chains on superconductors. *Nat. Commun.* **11**, 4707 (2020).
12. Balatsky, A. V., Vekhter, I. & Zhu, J.-X. Impurity-induced states in conventional and unconventional superconductors. *Rev. Mod. Phys.* **78**, 373–433 (2006).
13. Ruby, M. *et al.* Tunneling Processes into Localized Subgap States in Superconductors. *Phys. Rev. Lett.* **115**, 087001 (2015).
14. Klinovaja, J. & Loss, D. Composite Majorana fermion wave functions in nanowires. *Phys. Rev. B* **86**, 085408 (2012).
15. Peng, Y., Pientka, F., Glazman, L. I. & von Oppen, F. Strong Localization of Majorana End States in Chains of Magnetic Adatoms. *Phys. Rev. Lett.* **114**, 106801 (2015).
